# Implant prices and physician reimbursement have declined more than total costs and hospital payments in total shoulder arthroplasty

**DOI:** 10.1016/j.jseint.2026.101656

**Published:** 2026-02-10

**Authors:** Jonathan S. Yu, Charlotte Wahle, Kevin Heo, Tim Liu, Mathangi Sridharan, Nicole Newman-Hung, Frank Petrigliano, Andrew R. Jensen

**Affiliations:** a Department of Orthopedic Surgery, University of California Los Angeles, Los Angeles, CA, USA; b Department of Orthopedic Surgery, Emory University School of Medicine, Atlanta, GA, USA

**Keywords:** Total shoulder arthroplasty, Reverse total shoulder arthroplasty, Implant, Price, Cost, Reimbursement

## Abstract

**Background:**

Total shoulder arthroplasty (TSA) is among the fastest-growing joint replacement procedures in the United States. As bundled payment models and value-based care initiatives expand, surgeons and hospitals face increasing pressure to contain costs. Implants account for a substantial portion of procedural expenses, yet trends in TSA implant pricing relative to total costs, hospital and physician reimbursement, and patient out-of-pocket (OOP) spending remain poorly characterized.

**Methods:**

Implant prices for anatomic total shoulder arthroplasty (aTSA) and reverse total shoulder arthroplasty (rTSA) from 2010 to 2022 were obtained from the largest publicly available implant registry, Orthopedic Network News. Cost, reimbursement, and patient OOP spending data were sourced from a commercial insurance claims database. All costs, reimbursements, and prices were adjusted for inflation. Trends were analyzed using linear regressions.

**Results:**

A total of 59,442 TSA procedures were included. The average implant price was $5,928 for aTSA and $8,720 for rTSA. Between 2010 and 2022, implant prices declined substantially for both aTSA (−45.3%, *P* < .001) and rTSA (−42.9%, *P* < .001). Physician reimbursement decreased by 46.4% (*P* < .001). In contrast, overall costs and hospital reimbursement decreased more modestly (−32.3%, *P* = .06; −29.8%, *P* = .09, respectively). Patient OOP costs remained relatively unchanged (−13.9%, *P* = .25).

**Conclusion:**

Although implant prices have decreased substantially over time, the financial benefit has not been realized by patients or surgeons. This is the first study to contextualize implant cost trends alongside total costs, hospital reimbursement, physician reimbursement, and OOP patient expenses in TSA, highlighting persistent gaps in cost transparency. In an era of increasing TSA volumes and growing cost-control pressures, these findings underscore the need for surgeon awareness of implant costs, which will be particularly important in guiding future changes to clinical practice, payment models, and policy.

Total shoulder arthroplasty (TSA) is an increasingly common and cost-intensive procedure in the United States, with utilization projected to rise more rapidly than hip or knee arthroplasty over the coming decade.^3^^,^^13^ The aging population and expanding indications for reverse TSA have further accelerated demand, placing increased emphasis on cost containment while maintaining high-quality outcomes.^8^^,^^10^^,^^11^

Implants represent a substantial proportion of arthroplasty expenses.^1^ Despite their central role, implant prices remain opaque and are often unknown even to operating surgeons. Surveys have shown that most orthopedic surgeons significantly underestimate implant costs and demonstrate limited awareness of price differences across systems and vendors.^6^^,^^23^ This lack of cost awareness may hinder the ability of providers to make cost-conscious decisions or advocate for more efficient resource utilization.

In hip and knee arthroplasty, prior work has documented declining physician reimbursement and increasing financial pressure on hospitals, even as implant prices have begun to decrease.^1^^,^^21^^,^^31^ However, few studies have examined how these changes align with total costs, hospital reimbursement, and patient out-of-pocket (OOP) spending in TSA. Although TSA has been shown to be cost-effective, its value is highly sensitive to implant price thresholds.^8^^,^^9^ To date, no study has comprehensively contextualized implant pricing trends alongside overall costs, reimbursement, and OOP expenses in TSA. Because implants remain one of the most modifiable contributors to episode-of-care costs, understanding these trends is essential to inform both clinical practice and policy.

The purpose of this study was to evaluate inflation-adjusted trends in implant prices for anatomic and reverse TSA between 2010 and 2022. We compared these changes to trends in overall costs, hospital and physician reimbursement, and OOP spending using nationally representative datasets.

## Methods

### Data sources

We examined inflation-adjusted implant pricing trends and cost-related data for primary anatomic total shoulder arthroplasty (aTSA) and reverse total shoulder arthroplasty (rTSA) between 2010 and 2022. Implant pricing data were obtained from Orthopedic Network News (ONN), the largest publicly available orthopedic implant registry. Data on total procedural costs, hospital reimbursement, physician reimbursement, and patient OOP expenses were extracted from the IBM MarketScan Commercial Database, a large administrative claims database comprising employer-sponsored insurance claims across the United States. TSA procedures were identified using the single Current Procedural Terminology (CPT) code 23472, which encompasses both aTSA and rTSA.

### Cost adjustment and categorization

All costs, reimbursements, and implant prices were adjusted for inflation to 2022 US dollars using the Consumer Price Index. Costs were categorized into the following categories: (1) Overall costs: total expenditure per procedure, including facility and provider payments. (2) Hospital reimbursements: payment made by insurers to hospitals. (3) Physician reimbursements: payments made to healthcare providers performing the surgery. (4) OOP costs: direct patient contributions, such as copayments and deductibles. (5) Implant prices: average implant prices sourced from ONN.

### Statistical analyses

Descriptive statistics were used for patient demographics and overall costs, reimbursement, and payment, and are reported as mean and standard deviation or number with percentages. Trends were analyzed using linear regression and inflation-adjusted percent change between 2010 and 2022. Statistical significance was determined using a *P* value <.05. Subgroup analyses were performed for primary and revision procedures. Within each figure, significant changes in costs or reimbursements were identified by dashed trend lines, while solid trend lines indicated no significant change.

## Results

**Figure 1:**
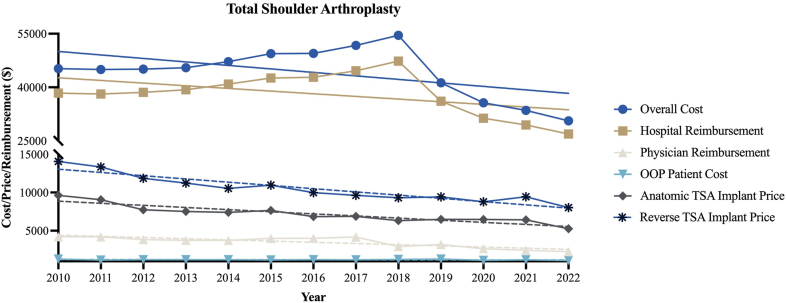
TSA cost, reimbursement, and implant price trends. *Dashed trendlines* represent significant change while *solid trendlines* represent no significant change. *TSA*, total shoulder arthroplasty; *OOP*, out-of-pocket.

Table I outlines the characteristics and cost breakdowns for aTSA and rTSA procedures from 2010 to 2022. A total of 59,442 TSA procedures were identified between 2010 and 2022. The average inflation-adjusted implant price was $5,928 for aTSA and $8,720 for rTSA. Figure 1 visualizes trends over time in total costs, hospital reimbursement, physician reimbursement, OOP costs, and implant prices for TSA, while Table II provides statistical summaries of the percentage changes, regression coefficients (β), and *P* values for each cost component. Over the study period, implant prices declined significantly by 45.3% for aTSA and 42.9% for rTSA (*P* < .001 for both). Physician reimbursement decreased by 46.4% (*P* < .001). In contrast, overall costs decreased by 32.3% (*P* = .06) and hospital reimbursement by 29.8% (*P* = .09). Patient OOP costs decreased by 13.9% (*P* = .25).

**Table I:** Characteristics and overall costs, reimbursement, and payment for TSA, 2010-2022.

Characteristics	Total shoulder arthroplasty
Total number of procedures	59,442
Age, mean (SD)	66.9 (10.0)
Female (%)	30,941 (52.1%)
Length of stay, mean (SD)	1.9 (2.4)
Total cost for procedure, $	$37,027
Total hospital charge, $	$31,878
Total physician payment, $	$3,016
Total OOP cost for procedure, $	$979
Average selling price for anatomic TSA implant from ONN, $	$5,982
Average selling price for reverse TSA implant from ONN, $	$8,720

*OOP*, out-of-pocket; *TSA*, total shoulder arthroplasty; *ONN*, Orthopedic Network News; *SD*, standard deviation.

**Table II:** Upper extremity trauma linear regression trends from 2012 to 2022.

Procedure	Category	Percent change from 2012 to 2022	Slope (95% CI)	*P* value
Total shoulder arthroplasty	Overall cost	−32.3%	−978.2 (−2006 to 50.04)	.06
	Hospital reimbursement	−29.8%	−745.8 (−1637 to 144.9)	.09
	Physician reimbursement	−46.4%	−155.5 (−217.5 to −93.39)	**<.001**
	OOP patient cost	−13.9%	−4.9 (−13.79 to 3.936)	.25
	Anatomic TSA implant price	−45.3%	−277.3 (−352.9 to −201.7)	**<.001**
	Reverse TSA implant price	−42.9%	−423.9 (−526.5 to −321.3)	**<.001**

*OOP*, out-of-pocket; *TSA*, total shoulder arthroplasty; *CI*, confidence interval.

*P*-values highlighted in bold represent significant decreases.

## Discussion

This study presents a comprehensive analysis of implant pricing trends in TSA alongside overall costs, hospital reimbursement, physician reimbursement, and patient financial burden. Between 2010 and 2022, implant prices decreased by more than 40% for both anatomic and reverse TSA, and physician reimbursement fell by nearly 50%. In contrast, overall costs and hospital reimbursement decreased only modestly and without statistical significance. These findings highlight a widening disconnect between declining implant prices and the financial realities faced by patients and surgeons.

Our results parallel findings in hip and knee arthroplasty. Recent analyses have demonstrated that implant prices and physician reimbursement in total knee arthroplasty and total hip arthroplasty have declined substantially more than total costs and hospital payments.^31^ Earlier work showed that implant prices historically rose steadily, often outpacing inflation and reimbursement,^1^^,^^17^ but more recent data suggest that hospital leverage and manufacturer competition have driven sustained reductions in implant prices across arthroplasty.^5^^,^^15^^,^^20^ Despite these decreases, overall procedural costs remain largely stable, with hospitals and payers capturing most of the financial benefit rather than patients or providers.^16^^,^^21^

Implants constitute one of the most modifiable components of arthroplasty costs, and their pricing dynamics are central to bundled payment and value-based care models.^5^^,^^26^ In lower extremity arthroplasty, initiatives such as the Comprehensive Care for Joint Replacement and Bundled Payments for Care Improvement have incentivized hospitals to pursue implant standardization and price capitation agreements.^14^^,^^22^^,^^26^^,^^29^ These same pressures likely extend to TSA, where implant costs are comparable to those of total hip and knee arthroplasty.^12^ However, our findings suggest that these system-level cost reductions have not translated into lower patient OOP expenses or preserved surgeon reimbursement, raising concerns about the equity of current cost-sharing structures.^4^^,^^18^^,^^24^

Another key issue is surgeon awareness of implant pricing. Multiple studies have demonstrated that orthopedic surgeons frequently underestimate implant costs, with limited ability to correctly identify relative differences across systems or vendors.^6^^,^^23^ This lack of transparency limits surgeons' ability to engage in cost-conscious decision-making and to advocate for policies that ensure savings are shared across stakeholders. Interventions such as providing implant price data on preference cards, incorporating pricing into residency curricula, and fostering collaboration with value analysis committees may help narrow this gap.^25^^,^^27^ Moreover, federal hospital price transparency regulations create opportunities to make implant pricing more accessible, though compliance and usability remain variable.^16^

The implications of these trends extend to both practice and policy. Physician reimbursement has continued to decline under frameworks such as the Medicare Access and Children's Health Insurance Program Reauthorization Act, despite increasing case complexity and rising procedural volumes.^18^^,^^24^^,^^26^ Hospitals may be benefiting disproportionately from vendor discounts and cost-control strategies, while surgeons face eroding reimbursement and patients experience relatively unchanged OOP costs. As TSA volumes rise in parallel with an aging population and expanded indications, these dynamics threaten long-term sustainability and raise questions about fairness for both providers and patients.

Surgeon awareness of implant costs has increasingly been recognized as a key determinant of financial stewardship in orthopedic care. Prior literature demonstrates that many orthopedic surgeons have limited knowledge of the actual prices of the devices they routinely implant, often due to confidentiality clauses, vendor contracting structures, and restricted access to institutional pricing data.^23^^,^^30^ This lack of transparency limits surgeons' ability to meaningfully participate in cost containment efforts and contributes to misaligned financial incentives. Importantly, studies have shown that when surgeons are provided with reliable cost information, they frequently select lower-cost implants within the same class, resulting in substantial reductions in implant expenditures without altering treatment strategy or compromising outcomes.^19^^,^^28^ These findings suggest that transparency initiatives may facilitate implant standardization and enhance hospital leverage in vendor negotiations. However, physician reimbursement is largely determined by payer fee schedules, and patient OOP expenses are influenced by insurance benefit design; therefore, declines in implant pricing alone do not necessarily translate into financial benefit for either surgeons or patients. This structural disconnect likely contributes to the observed divergence between decreasing implant prices and continued reductions in surgeon reimbursement.

These results also support a growing emphasis on formal cost-awareness education and surgeon engagement in institutional decision-making. Structured educational interventions have been shown to reduce variation in implant and supply utilization and to produce measurable decreases in procedural expenditures.^2^ Practical, implementable strategies include incorporating routine pricing transparency into surgeon education, standardizing preference cards, and increasing orthopedic representation within hospital value-analysis committees and procurement discussions.^7^ Although such initiatives may not directly influence payer-driven reimbursement models, they may improve alignment between surgeons and hospitals, promote more equitable cost-sharing frameworks, and strengthen advocacy efforts for sustainable compensation models. In an increasingly value-focused healthcare environment, surgeon familiarity with implant economics and active participation in institutional cost governance remain essential for ensuring that cost reductions are achieved responsibly while maintaining access to high-quality surgical care.

This study has limitations. First, implant price data were obtained from ONN, which reports average selling prices from a sample of hospitals and vendors and may not capture all institutional or regional variation. Second, reimbursement and cost data were derived from the MarketScan Commercial Claims and Encounters database, which represents patients with employer-sponsored private insurance and may not generalize to Medicare, Medicaid, or uninsured populations. Third, because implant and reimbursement data came from separate sources, patient-level linkage was not possible. Finally, changes in implant utilization patterns, surgical technique, and patient demographics may also have contributed to observed trends.

## Conclusion

Over the past decade, implant prices for both anatomic and reverse TSA have declined substantially, accompanied by a similar decrease in physician reimbursement. In contrast, overall procedural costs and hospital reimbursements decreased only modestly, while patient OOP expenses remained largely unchanged. These trends underscore the importance of cost transparency and raise critical questions about the equity of current cost-sharing structures. They also emphasize the need for continued collaboration among payers, hospitals, and policymakers to better understand and address the impact of shifting cost dynamics, particularly as TSA volumes grow and value-based care increasingly prioritizes cost-effectiveness.

## Disclaimers

Funding: No funding was disclosed by the authors.

Conflicts of interest: Andrew R. Jensen is a consultant for Zimmer Biomet. Frank Petrigliano is a consultant for Exactech. Any additional authors, their immediate families, and any research foundations with which they are affiliated have not received any financial payments or other benefits from any commercial entity related to the subject of this article.

## Data Availability

Analysis and other materials used in this study are available upon request to the authors.
